# Anti-asthmatic miR-224-5p inhibits the FHL1/MAPK pathway to repress airway smooth muscle cell proliferation in a murine model of asthma-like airway inflammation

**DOI:** 10.1186/s13223-022-00724-9

**Published:** 2022-10-02

**Authors:** Zhifang Zhuang, Yanjuan Zhou, Jiao Xu, Leying Pan

**Affiliations:** 1 Wujin Hospital Affiliated with Jiangsu University, Changzhou, 213002 People’s Republic of China; 2 Wujin Clinical College of Xuzhou Medical University, Changzhou, 213002 People’s Republic of China

**Keywords:** MicroRNA-224-5p, Four-and-a-half LIM protein 1, MAPK pathway, Airway inflammation, Asthma, Airway smooth muscle cells

## Abstract

**Background:**

The proliferation of airway smooth muscle cells (ASMCs) contributes to the contractility and inflammation in the pathophysiology of asthma. This intrigued us to clarify the effect of microRNA (miR)-224-5p on biological characteristics of ASMCs in mice with asthma-like airway inflammation and responses through the FHL1-dependent MAPK pathway.

**Methods:**

An ovalbumin (OVA)-induced asthma mouse model was established, where ASMCs were isolated. The expression of FHL1 was determined in asthmatic mice. Artificial modulation of FHL1 expression was performed to explore its effect on airway inflammation of asthmatic mice and ASMC proliferation and apoptosis. Afterwards, we analyzed the interaction among miR-224-5p, FHL1 and the MAPK pathway, and explored their combined impacts on airway inflammation of asthmatic mice and ASMC proliferation and apoptosis.

**Results:**

FHL1 was highly expressed and miR-224-5p was poorly expressed in asthmatic mice. FHL1 was verified to be a target of miR-224-5p. Loss of FHL1 function reduced airway inflammation in asthmatic mice and proliferation of ASMCs while inducing their apoptosis. Besides, miR-224-5p inhibited the MAPK pathway by binding to FHL1. Overexpression of miR-224-5p relieved airway inflammation, inhibited ASMC proliferation, and increased apoptosis, which could be reversed by overexpression of FHL1.

**Conclusion:**

Altogether, miR-224-5p inhibited airway inflammation in asthmatic mice and ASMC proliferation through blocking the MAPK pathway by down-regulating FHL1.

**Supplementary Information:**

The online version contains supplementary material available at 10.1186/s13223-022-00724-9.

## Background

Asthma is one of the most prevalent airway inflammatory diseases [1], characterized by respiratory symptoms, intermittent airway obstruction and airway hyper-responsiveness due to airway inflammation and remodeling [2, 3]. Evidence has been presented demonstrating the involvement of airway smooth muscle cells (ASMCs) in the airway hyperresponsiveness as well as the inflammatory reactions and remodeling in asthma [4, 5], the proliferative and migratory potential of which is a major hallmark of the asthmatic pathogenesis [6, 7]. Further explorations are warranted to clarify the mechanistic basis behind migratory capacity of ASMCs, and to validate if repression of this migratory potential would offer a therapeutic possibility for asthma management [8, 9].

It is interesting to note that four-and-a-half LIM domain 1 (FHL1), is a member of the FHL subfamily [10], characterized by an N-terminal half LIM domain, the downregulation of which inhibits proliferation of pulmonary artery SMCs [11, 12]. In addition, an isoform of FHL1, KyoT2, can be considered as a potential target for the treatment of asthma since its overexpression downregulates airway remodeling [13] and resistance in asthmatic mice [14]. Interestingly, FHL1 has been involved in various cell pathways including estrogen- [15], Notch- 16, and MAPK [17] cascades. It has also been highlighted previously that FHL1 senses the biomechanical stress-induced responses via the MAPK pathway [18]. Interestingly, MAPK pathway activation is associated with those inflammatory responses of patients with asthma [19]. Accumulating evidences have confirmed that p38MAPK pathway activation is responsible for the IFN-γ release in lymphocytes in asthma, contributing to asthmatic inflammation [20, 21]. Hence, we speculated that FHL1 could mediate the MAPK pathway to affect the airway inflammation in asthma.

MicroRNAs (miRNAs) belong to the class of small non-coding RNA molecules and have been identified as important regulators of target genes of some pathways [22]. miRNAs controlling some target genes are important for inflammatory responses and development of asthma [19]. Strikingly, miR-224 is reported to be expressed at a low level in asthmatic patients [23]. Moreover, overexpression of miR-224 is capable of suppressing airway epithelial cell inflammation and airway remodeling in PM2.5-induced asthmatic mice by decreasing the expression of TLR2 [24].

In our study, FHL1 was predicted to be a target of miR-224-5p based on the results from several databases (microRNA, TargetScan, RNA22, and starBase). Given the aforementioned findings, we performed the current study aiming to ascertain the effect of miR-224-5p on the airway inflammation in asthma, which involved FHL1 and the MAPK pathway (Additional file 1: Fig. S1).

## Materials and methods

### Ethics statement

This study was approved by the Institutional Animal Care and Use Committee of the Wujin Hospital Affiliated with Jiangsu University, and carried out in strict accordance with the recommendations in the Guide for the Care and Use of Laboratory Animals published by the US National Institutes of Health.

### Microarray profiling

Two asthma-related gene expression datasets (GSE27066 and GSE6858) were retrieved from the GEO database, followed by differential expression analysis. Both of the datasets contained gene expression data from lung tissues of ovalbumin (OVA)-induced asthmatic mice and control mice. The annotated platforms of GSE27066 and GSE6858 datasets were GPL1261-[Mouse430_2] Affymetrix Mouse Genome 430 2.0 Array. Differentially expressed genes (DEGs) were screened using limma package of R language [15], with threshold values set as *p* < 0.05 and |Log FoldChange (FC)|> 2. Then a heatmap of DEGs was plotted. Venn online analysis tool was employed to calculate and plot customized Venn diagrams for comparison of DEGs in the two datasets and determination of asthma-related DEGs. Afterwards, microRNA, TargetScan, RNA22 and starBase databases were applied to predict the miRNAs that regulated FHL1, followed by construction of miRNA-mRNA regulatory network.

### Murine asthma-like airway inflammation model establishment and animal grouping

A total of 90 male or female BALB/c mice (weighing 13–18 g and aged 7 weeks) were provided by Animal Core Facility of Jiangsu University (Jiangsu, China). Among them, 80 mice were selected for asthma-like airway inflammation modeling and the other 10 was set as normal control. Each mouse was intraperitoneally injected with 200 μL sensitization solution (20 μg OVA and 150 μL aluminum hydroxide adjuvant) at days 0 and 14 [16]. Additionally, these mice were treated with 1% OVA by daily intranasal inhalations from day 21 to day 55 (20 min each time). During the operation, the activity and respiration of mice were observed. Besides, the remaining 10 mice were treated with autoclaved phosphate-buffered saline (PBS; pH = 7.4) instead as normal control.

Mice in the normal group had mild symptoms such as scratching, sneezing or cough, but mice after sensitization operations exhibited asthma-like symptoms such as irritability, scratching, sneezing or coughing, and shortness of breath. As inhalations increased, these symptoms were aggravated. Meanwhile, these mice were found arched or lying motionless with symptoms including cyanosis in nose and mouth, ears and toes, decreased appetite, polycopria, thin sloppy stool, as well as lackluster hair, indicating the successful model establishment.

After anesthesia, successfully modeled asthmatic mice were grouped (n = 10) including an asthma group wherein mice remained untreated and other treatment groups subjecting to administration with lentivirus carrying overexpression (oe)-FHL1, short hairpin RNA (sh)-FHL1, miR-224-5p-agomir, miR-224-5p-agomir + oe-NC, or miR-224-5p-agomir + oe-FHL1, or the negative control (NC) (oe-NC, sh-NC or NC-agomir). The corresponding lentivirus was intranasally administered into mice at a titer of 5 × 10^7^ TU/mL (200 µL per mouse) from day 26 once a week until day 47 [25]. The lentiviral vectors were purchased from Shanghai GenePharma (Shanghai, China).

The mice were euthanized on day 55 after the successful establishment of asthma-like airway inflammation. The bronchoalveolar lavage fluid (BALF) in the right lung was obtained by intratracheal infusion. Then the BALF was centrifuged (3000 r/min) for 10 min whereupon the supernatant was collected and stored at − 70 °C for further use. The left lung of mice was made into paraffin-embedded slices for hematoxylin–eosin (HE) staining. The flow chart of in vivo experiments is shown in Additional file 2: Fig. S2.

### Assessment of airway responsiveness

Mice were anesthetized with pentobarbital sodium on day 55. The mice were then subjected to trachea cannula with connection to a breathing machine and atomization device along with Buxco spirometer, by which the airway responsiveness of mice was measured on the 1^st^, 3^rd^ and 7^th^ day. Subsequently, the trachea was exposed and cannulated to a small-animal ventilator controlled by computer. Next, the mice were ventilated with a tidal volume of 10 mg/kg at a frequency of 150 breaths/min and a positive end-expiratory pressure of 2 cm H_2_O to reach a mean lung volume, which is close to that during spontaneous breathing. Each mouse was exposed to PBS and then to increasing concentrations of methacholine (Mch) in PBS (0, 10, 30, 100, and 200 mg/mL). The peak airway responses to the inhaled Mch were recorded. With Mch as the activator, the inspiratory resistance (RI) caused by two-fold dosage of Mch (0.39–200 g/L) was examined until the original RI value was doubled. The dosage of Mch was switched into logarithm (PC_100_) for statistical analysis. Higher PC_100_ value represented lower airway reactivity [26, 27].

### Immunohistochemistry

Paraffin-embedded tissue blocks of left lung were sectioned into 4-μm tissue slices, which were treated with 3% alcohol and hydrogen peroxide (H_2_O_2_). After antigen retrieval and goat serum blocking, the slices were incubated with rabbit monoclonal antibody to FHL1 (1: 500; ab255828; Abcam, Cambridge, UK), rabbit monoclonal antibody to vascular endothelial growth factor (VEGF; 1: 250; ab32152; Abcam), rabbit monoclonal antibody to p-ERK1/2 (1: 100; ab214362; Abcam), rabbit monoclonal antibody to p-p38MAPK (1: 500, ab47363; Abcam) and rabbit monoclonal antibody to p-JNK (1: 100; ab124956; Abcam) overnight at 4 °C, and then re-probed with the secondary antibody, horseradish peroxidase (HRP)-labeled goat anti-rabbit immunoglobulin G (IgG) H&L (1: 1000; ab6721; Abcam) at 37 °C for 20 min. Subsequently, the slices were supplemented with OVA solution (Hebei Bio-high Technology, Shijiazhuang, Hebei, China) at 37 °C for 20 min. The slices were then counterstained by hematoxylin for 1 min, hydrolyzed by 1% hydrochloric acid ethanol, and observed under the light microscope. Five high-power visual fields were randomly selected for each slice selects with 100 cells per field. The cells stained to be brown were regarded as positive cells, and the percentage of positive cells was counted.

### HE staining

Prepared sections of mouse lung tissues were stained with hematoxylin (Solarbio, Beijing, China) for 2 min. Afterwards, the slices were rinsed by distilled water for 1 min and stained by eosin for 1 min. At last, pathological changes of the slices were observed under a microscope (XP-330, Bingyu Optical Instrument, Shanghai, China).

### Masson’s trichrome staining

The lung tissue sections were stained successively with Regaud’s hematoxylin dye solution for 5—10 min and Masson’s dye solution for 5—10 min. After being immersed in 2% acetic acid solution, sections were hydrolyzed by 1% molybdate solution. Following that, the slices were stained with aniline blue and aqua anion dye liquor, and observed under a microscope. At last, collagen deposition in lung tissues was observed under the microscope. Collagen fibers were stained to be blue, the cytoplasm of muscle fibers stained to be purplish red, and the nucleus stained to be blue brown [28]. NIH ImageJ image analysis software was used to quantitatively analyze the collagen deposition area. The percentage of collagen deposition area = collagen fiber/tissue area × 100%.

### BALF cell counting

The BALF pellets were resuspended with 0.5 mL precooled and sterile PBS. Then total cell number was measured with 0.1 mL BALF by a blood cell count plate and 0.2 mL diluted suspension was dropped in the clean slide. Wright's-Giemsa staining solution A was dropped to the slide. The slide was stained with Wright's-Giemsa staining solution B for 10 min. On the basis of cell morphology and color, 300 white blood cells were tested for cell counting. Also, the numbers of total cells and eosinophils in BALF were observed under an optical microscope.

### ELISA

The BALF supernatant of mice was collected to determine the levels of interleukin (IL)-4 (18.75 pg/mL—1200 pg/mL, ab221833), IL-5 (2.05 pg/mL—500 pg/mL, ab100711), IL-13 (1.95 pg/mL—125 pg/mL, ab219634) and IFN-γ (2.74 pg/mL—2000 pg/mL, ab100689) using ELISA kits (Abcam).

### Isolation and identification of primary ASMCs

The mice were euthanized with intraperitoneal anesthesia (0.1 mL/20 g) of the mixture of ketamine and droperidol at a ratio of 1: 1. After being sterilized with 75% alcohol, the chest was cut open to isolate the trachea and lung tissues, which were put into the D-Hanks’s solution containing 100 U/mL mycillin at 4 °C.

The tissue blocks (≤ 1 mm) were transferred into a 15 mL centrifuge tube, and detached with 2 mL prepared collagenase type I solution containing 2 mg/mL collagenase type I, 2.5 mg/mL papain and 2.5 mg/mL bovine serum albumin solution, and D-Hanks’s solution in a 5% CO_2_ incubator for 25 min at 37 °C. DMEM medium supplemented with high glucose and FBS was applied to stop the detachment. Then the tissue blocks were centrifuged at 178 × g for 5 min. After the supernatant was discarded, the tissue blocks were detached with 2 mL 0.25% trypsin and 0.02% EDTA solution again for 15 min, followed by centrifugation. At last, the tissue blocks were added with 1.5 mL 20% DMEM with high glucose containing FBS for semi-open incubation and then further cultured with replaced solution after 3 d. Immunohistochemical streptavidin–biotin-peroxidase (SP) staining for ASMC-specific α-actin presented positive to identify ASMCs. The morphology of ASMCs incubated at the 3^rd^ day and 8^th^ day was observed under an inverted microscope.

### Cell transduction

The ASMCs at passage 3 were trypsinized and seeded in a 24-well plate. Cells were assigned into normal (ASMCs isolated from normal mice), asthma (ASMCs isolated from asthmatic mice), NC-mimic, miR-224-5p mimic, NC-inhibitor, miR-224-5p inhibitor, sh-NC, sh-FHL1, oe-NC, oe-FHL1, miR-224-5p mimic + oe-NC, and miR-224-5p mimic + oe-FHL1 groups. Upon reaching 30–50% cell confluence, cells were transduced with corresponding plasmids using Lipofectamin 2000 reagents (11,668–019, Invitrogen, CA) and then incubated for 6—8 h. The medium was renewed with complete medium for another incubation for 24—28 h. Plasmids were constructed by Sangon Biotech (Shanghai, China).

### Dual-luciferase reporter assay

On the basis of bioinformatically predicted miR-224-5p binding site in FHL1 mRNA 3’-untranslated region (3’-UTR), the wild type (WT) and mutant (mut) sequences were designed and synthesized with the use of Xho I and Not I double restriction-enzyme digestion. The synthesized fragments were cloned into the PUC57 vector, which was then identified using DNA sequencing assay, and sub-cloned to the psiCHECK-2 vector. Next, plasmids were transferred into Escherichia coli DH5α to amplify the plasmid. The plasmids were extracted using Omega plasmid miniprep kit (D1100-50 T, Solarbio). Based on protocols of a dual luciferase assay kit (D0010, Solarbio), the luciferase intensity was determined by a Glomax20/20 luminometer (E5311, Zhongmei Biotechnology, Xi’an, Shaanxi, China).

### Reverse transcription-quantitative polymerase chain reaction (RT-qPCR)

TRIzol reagents (15,596–018, Solarbio) were applied to extract total RNA from lung tissues and transfected ASMCs. Primers of miR-224-5p, FHL1, ERK, p38MAPK and JNK were designed and synthesized by Takara (Dalian, China) (Additional file 4: Table S1). The extracted RNA was reverse transcribed into complementary DNA (cDNA) utilizing the PrimeScript RT kit (RR036A, Takara, Tokyo, Japan). The fluorescence quantitative PCR operation was subsequently carried out as per the instructions of the SYBR® Premix Ex TaqTM II kit (RR820A, TaKaRa, Tokyo, Japan) on an ABI 7500 instrument (Applied Biosystems, Foster City, CA). As normalized to U6 or GAPDH, relative expression was determined by the quantitative method 2^−△△Ct^.

### Western blot analysis

Total protein was extracted from mouse left lung tissues or ASMCs using a RIPA kit (R0010, Solarbio), with the concentration measured by a bicinchoninic acid (BCA) kit (G3522-1, GBCBIO Technologies, Guangzhou, Guangdong, China). The proteins were then wet-transferred onto polyvinylidene fluoride (PVDF) membranes after polyacrylamide gel electrophoresis separation. After 1-h blocking with 5% BSA, the membranes were then incubated with the following diluted primary antibodies: rabbit monoclonal antibody to FHL1 (1: 1000; ab255828; Abcam), rabbit monoclonal antibody to p-ERK1/2 (1 µg/ml; 1: 100; ab184699; Abcam), mouse monoclonal antibody to p38MAPK (1: 1000; ab31828 Abcam), rabbit monoclonal antibody to JNK (1: 1000; ab179461; Abcam), rabbit monoclonal antibody to p-ERK1/2 (1: 100; ab184699; Abcam), rabbit monoclonal antibody to p-p38MAPK (1: 500, ab47363; Abcam), rabbit monoclonal antibody to p-JNK (1: 100; ab124956; Abcam), rabbit monoclonal antibody to cleaved PARP (1: 1000; ab32064; Abcam), and rabbit monoclonal antibody to cleaved caspase-3 (1: 1000; ab32042; Abcam) overnight at 4 °C. Afterwards, the membrane was incubated with the HRP-labeled secondary antibody, goat anti-rabbit (1: 5000; ab6721; Abcam) or goat anti-mouse (1: 5000; ab205719; Abcam) antibody to IgG. Blots were visualized with enhanced chemiluminescent (ECL) solution (WBKLS0500, Pierce Biotechnology Inc., Rockford, IL) and quantified by the ImageJ 1.48u software as normalized to GAPDH.

### MTT assay

Cells were collected after 48-h transfection and seeded into a 96-well plate (3 × 10^3^–6 × 10^3^ cells/well, 0.1 mL/well). Three time points at 24 h, 48 h and 72 h were set for the following experiments. Each well was added with 20 μL prepared MTT solution (5 mg/mL) and incubated at 37 °C for 2 h. Subsequently, a total of 150 μL DMSO was added into each well and optical density (OD) values of each well at 490 nm were detected using a microplate reader (NYW-96 M, Beijing Nuoyawei instrument, Beijing, China).

### Flow cytometry

Transfected cells were centrifuged and re-suspended in PBS to a concentration of 1 × 10^5^ cells/mL, and fixed with 1 mL 75% alcohol (pre-cooled at 20 °C) at 4 °C for 1 h. Next, cells were added with 100 μL RNase A, and uniformly mixed with 400 μL propidium iodide (PI, Sigma-Aldrich, St Louis, MO) for staining in the dark at 4 °C for 30 min. After that, flow cytometry was applied to record cell cycle of red fluorescence at a wavelength of 488 nm.

According to the protocols of an Annexin V-FITC cell apoptosis detection kit (Sigma-Aldrich, St Louis, MO), the Annexin-V-FITC/PI staining solution was prepared. Each 100 μL of staining solution was used to uniformly re-suspend 1 × 10^6^ cells by oscillating. After that, cells were incubated at room temperature for 15 min and mixed with 1 mL HEPES buffer. At last, 525 nm and 620 nm band-pass filter excited at the wavelength of 488 nm was applied to detect FITC, PI fluorescence and cell apoptosis.

### Statistical analysis

All data were processed by SPSS 21.0 statistical software (IBM Corp., Armonk, NY). The measurement data were expressed as mean ± standard deviation. The comparisons between two groups were analyzed by independent sample *t*-test, and the comparisons among multiple groups were conducted by one-way analysis of variance (ANOVA), followed by a Tukey multiple comparisons posttest. Data among multiple groups at various time points were analyzed by two-way ANOVA with Bonferroni corrections. *p* < 0.05 was considered statistically significant.

## Results

### FHL1 is highly expressed in mice with asthma-like airway inflammation

**Fig. 1:**
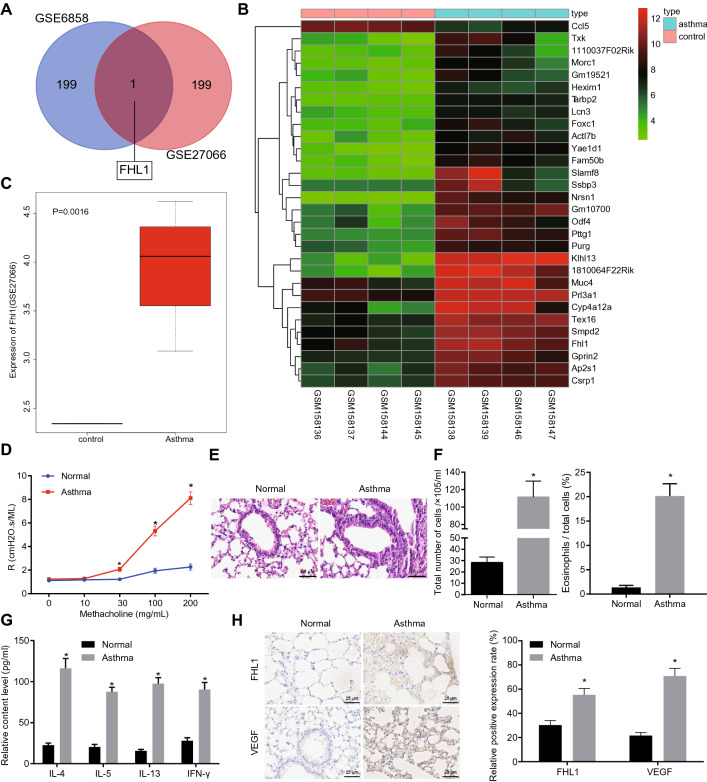
FHL1 expresses highly in mice with asthma-like airway inflammation. **A** The Venn map showing intersection of the top 200 DEGs obtained from asthma-related gene expression datasets GSE27066 and GSE6858. **B** The heatmap of the top 30 DEGs obtained from the GSE6858 dataset. The X axis represents the sample number, and the Y axis represents the DEGs; the upper right histogram is the color grade, where each rectangle corresponds to a sample expression value; the red indicates the high expression, and the green indicates the low expression. **C** The expression of FHL1 in asthma in the GSE27066 dataset. **D** Determination of airway responsiveness in mice with asthma-like airway inflammation. **E** HE staining images of bronchial cavities of similar diameter in lung tissues from normal and asthmatic mice. **F** The number of total cells and percentage of eosinophils in BALF of normal and asthmatic mouse lung tissues. **G** Levels of IL-4, IL-5, IL-13, and IFN-γ in BALF supernatant of normal and asthmatic mouse lung tissues measured by ELISA. **H** Immunohistochemical images and quantitative analysis of FHL1 and VEGF positive expression in normal and asthmatic mouse lung tissues. * *p* < 0.05 compared with normal mice. n = 10 mice for each treatment

Through profiling of asthma-related gene expression datasets GSE27066 and GSE6858, the top 200 DEGs of the two datasets were identified, respectively. Through the intersection, there was only one gene (FHL1) in the Venn diagram (Fig. 1A), highlighting FHL1 as an asthma-associated gene. A heatmap was then plotted based on the expression of top 30 asthma-related DEGs obtained from the GSE6858 dataset (Fig. 1B), which displayed higher FHL1 expression in the asthma samples than that in the control samples. FHL1 was also found to be abnormally overexpressed in asthma samples in the GSE27066 dataset (Fig. 1C).

In established asthmatic mice, an increase in airway responsiveness was observed when Mch exceeded 30 mg/mL (Fig. 1D). The bronchial lumen of similar diameters in the lung tissue sections was subjected to HE staining. The results (Fig. 1E) illustrated that the asthmatic mice presented with thicker airway epithelium and airway smooth muscle (ASM), and inflammatory cell infiltration under the mucous membrane. In addition, compared with the normal mice, the percentages of total inflammatory cells and eosinophils were increased in the BALF of asthmatic mice (Fig. 1F).

Next, the secretion of inflammatory factors, as reflected by levels of IL-4, IL-5, IL-13 and IFN-γ in the BALF supernatant determined by ELISA, was increased in asthmatic mice relative to normal mice (Fig. 1G). Immunohistochemistry analysis demonstrated that the positive rates of FHL1 and VEGF proteins were increased in lung tissues of asthmatic mice (Fig. 1H).

The aforementioned results suggested that the mouse asthma models were successfully established, where FHL1 exhibited a high expression.

### MiR-224-5p targets and downregulates FHL1 in ASMCs

**Fig. 2:**
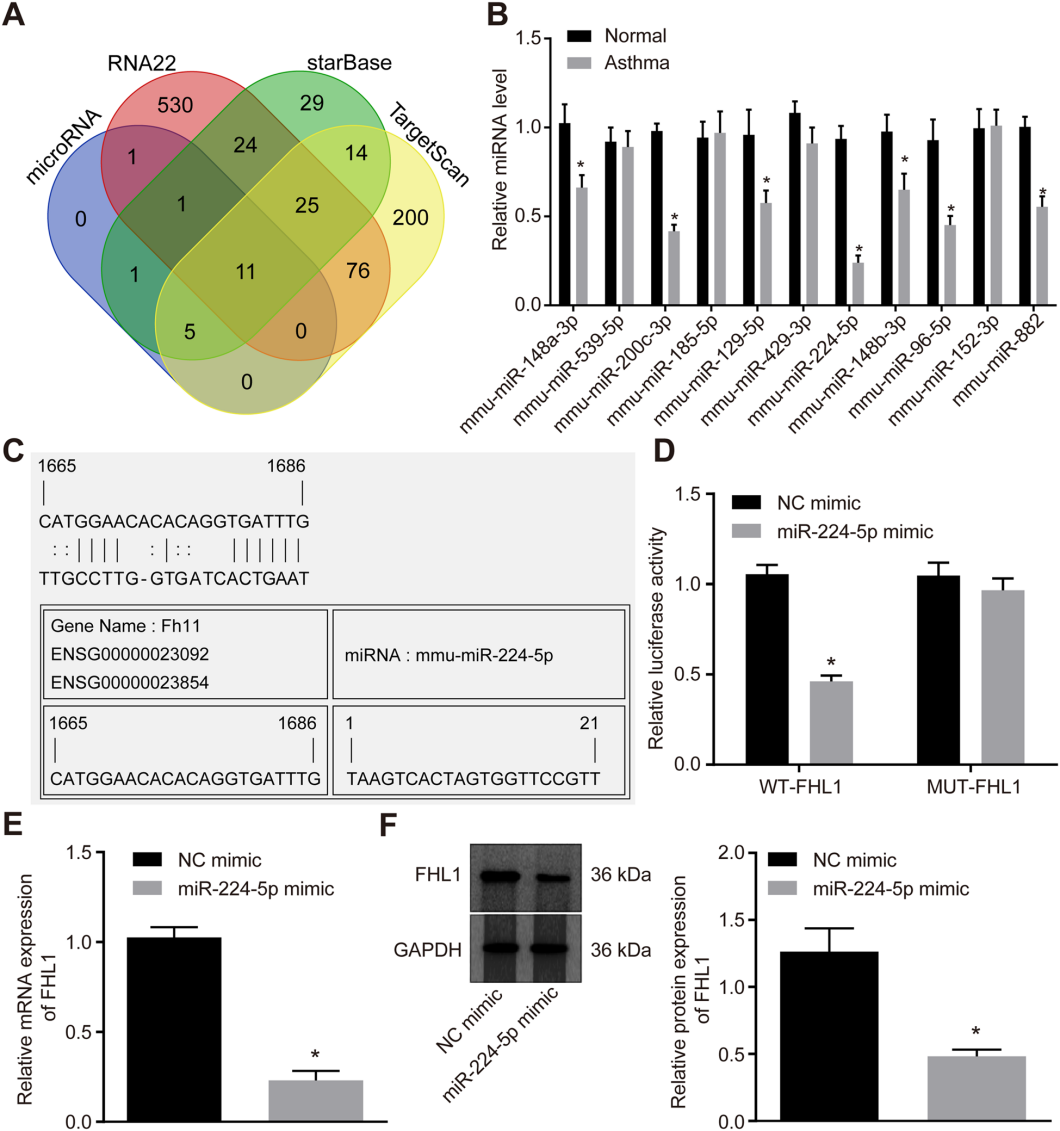
MiR-224-5p targets FHL1. **A** Venn map of predicted regulatory miRNAs of FHL1. Four ovals indicate the predicated results from the microRNA, TargetScan, RNA22 and starBase databases and the middle part indicates the intersection miRNAs. **B** Expression of the 11 predicted miRNAs in normal and asthmatic samples determined by RT-qPCR (n = 10). **C** Putative miR-224-5p binding sites in the 3’UTR of FHL1 mRNA. **D** The binding of miR-224-5p to FHL1 verified by dual-luciferase reporter gene assay. **E** FHL1 mRNA level in ASMCs isolated from asthmatic mice determined by RT-qPCR in the presence of miR-224-5p mimic. **F** Western blot analysis of FHL1 protein expression in ASMCs isolated from asthmatic mice in the presence of miR-224-5p mimic. * *p* < 0.05 compared with normal samples or NC-mimic. Cell experiments were performed in triplicate

MicroRNA and other databases were utilized to predict the upstream miRNAs that regulate FHL1, which were intersected to obtain the 11 miRNAs (Fig. 2A). Of note, among the 11 candidates miR-224-5p has been reported to be expressed poorly in asthma [23]. Additionally, RT-qPCR (Fig. 2B) identified the downregulation of miR-224-5p in asthma at a highest fold change among the 11 miRNAs. Thus, miR-224-5p was selected for following experiments.

Besides, the presence of a miR-224-5p-FHL1 binding site was predicted (Fig. 2C), which was further verified by dual-luciferase report gene assay (Fig. 2D). The luciferase activity of WT-FHL1 was inhibited by miR-224-5p mimic, but that of mut-FHL1 remained unaffected. Furthermore, miR-224-5p restoration resulted in decreased FHL1 expression in isolated mouse ASMCs (Fig. 2E, F).

To sum up, FHL1 was negatively regulated by miR-224-5p in ASMCs from asthmatic mice.

### Knockdown of FHL1 alleviates airway inflammation in mice with asthma-like airway inflammation

**Fig. 3:**
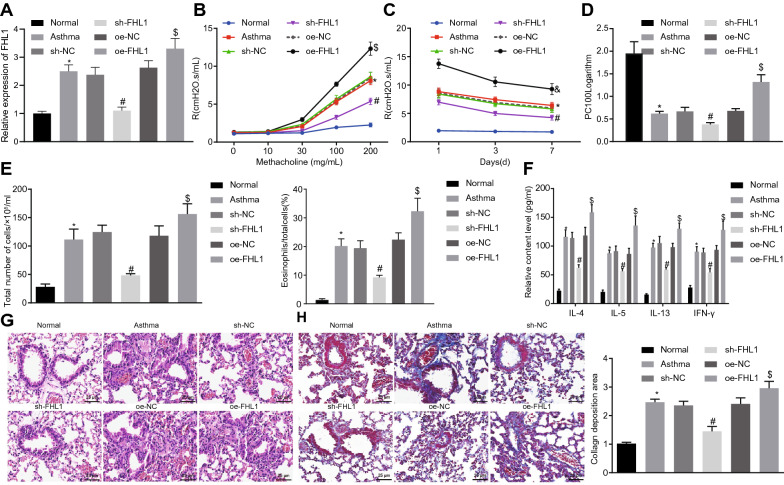
FHL1 down-regulation alleviates airway inflammation in mice with asthma-like airway inflammation. Normal mice served as the control and asthmatic mice were treated with lentivirus harboring oe-FHL1/oe-NC or sh-FHL1/sh-NC. **A** Expression of FHL1 in lung tissues of asthmatic mice determined by RT-qPCR. **B**, The airway resistance in asthmatic mice on the 1st day. **C** The airway resistance in asthmatic mice at various time points in response to Mch at a dosage of 200 mg/mL. **D** The airway responsiveness in asthmatic mice. **E** The number of total cells and percentage of eosinophils in BALF of asthmatic mice. **F** Levels of IL-4, IL-5, IL-13 and IFN-γ in BALF supernatant of asthmatic mice measured by ELISA. **G** HE staining images of lung tissues of asthmatic mice. **H** Masson’s trichrome staining images of lung tissues and collagen deposition area of asthmatic mice. * *p* < 0.05 compared with normal mice. # *p* < 0.05 compared with asthmatic mice treated with lentivirus harboring sh-NC. $ *p* < 0.05 compared with asthmatic mice treated with lentivirus harboring oe-NC. n = 10 mice for each treatment

We then manipulated FHL1 expression in asthmatic mice, and validated that oe-FHL1 successfully upregulated FHL1 and sh-FHL1 reduced FHL1 expression (Fig. 3A). In terms of airway responsiveness assessment, FHL1 knockdown lowered airway resistance, and forced FHL1 expression enhanced airway resistance in response to Mch was over 30 mg/mL (Fig. 3B). When the dosage of Mch reached 200 mg/mL, all asthmatic mice displayed higher airway resistance on the 1^st^ day than on the 7^th^ and 14^th^ day. Besides, asthmatic mice presented with higher airway resistance and lower PC _100_ logarithms than normal mice. The increases in airway resistance and airway reactivity were enhanced in response to FHL1 overexpression yet repressed by FHL1 silencing (Fig. 3C–D), suggesting that silencing of FHL1 suppressed airway response activity.

Moreover, it was also revealed that the number of total cells and the percentage of eosinophils in the BALF as well as levels of IL-4, IL-5, IL-13 and IFN-γ in the BALF supernatant of asthmatic mice were attenuated by FHL1 knockdown, all of which were increased in asthmatic mice overexpressing FHL1 (Fig. 3E–F).

As shown by HE staining and Masson’s trichrome staining in Fig. 3G–H, the asthmatic mice exhibited obviously augmented inflammatory cell infiltration (mainly eosinophils and lymphocytes) around the airway mucosa and blood vessel wall, thickened airway wall and ASM, increased folds of the mucosal epithelium, narrowed lumen partly blocked by mucus plugs, and increased staining area around bronchus and content of lung collagen. However, the asthmatic mice with sh-FHL1 exhibited orderly arranged epithelium of airway mucosa, slight damage of lung tissues, and reduced thickness of bronchial wall and content of collagen. The lung tissue injury of asthmatic mice was aggravated after overexpression of FHL1.

Overall, FHL1 downregulation could relieve airway inflammation and ameliorate lung tissue damage in the asthmatic mice.

### Silencing of FHL1 inhibits proliferation and induces apoptosis of ASMCs

**Fig. 4:**
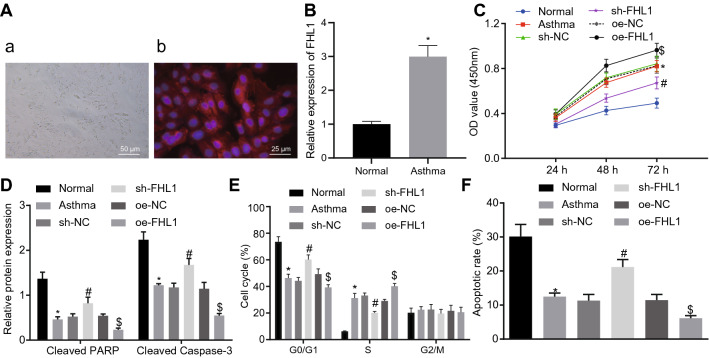
Knockdown of FHL1 induces inhibition of ASMC proliferation and promotion of their apoptosis in vitro. ASMCs isolated from normal mice remained non-transduced while ASMCs from asthmatic mice were transduced with oe-FHL1/oe-NC or sh-FHL1/sh-NC. **A** ASMCs from asthmatic mice identified by immunofluorescence. Morphological characteristics (a) and fluorescence microscopic observation (b) of ASMCs. **B** FHL1 mRNA expression in ASMCs determined by RT-qPCR. **C**, ASMC viability assessed by MTT assay. **D** Protein levels of cleaved PARP and cleaved caspase-3 in ASMCs measured by Western blot analysis. **E** Flow cytometric data showing cell cycle distribution. **F** Flow cytometric data showing apoptosis of ASMCs. * *p* < 0.05 compared with ASMCs from normal mice. # *p* < 0.05 compared with asthmatic mice-derived ASMCs transfected with sh-NC. $ *p* < 0.05 compared with asthmatic mice-derived ASMCs transfected with oe-NC. Cell experiments were performed in triplicate

ASMCs were isolated from asthmatic mice to investigate the effect of FHL1 on ASMCs. The isolated cells were spindle-shaped, presented abundant cytoplasm and centered nucleus, and high-density cells presented partially overlapping peak (Fig. 4A), suggesting that the isolated cells were ASMCs. Higher expression of FHL1 mRNA was observed in ASMCs derived from asthmatic mice (Fig. 4B).

MTT assay, flow cytometry, and Western blot analysis subsequently unveiled enhanced viability of ASMCs, fewer cells arrested at the G1/G0 phase, more cells arrested at S phase, decreased cell apoptosis, and reduced proteins levels of cleaved PARP and cleaved caspase-3 in the ASMCs from asthmatic mice, which were reversed by silencing of FHL1 but intensified in response to forced FHL1 expression (Fig. 4C–F).

Taken together, loss of FHL1 function impaired ASMC viability and promoted their apoptosis.

### MiR-224-5p alleviates airway inflammation and lung tissue damage in asthmatic mice by targeting FHL1

**Fig. 5:**
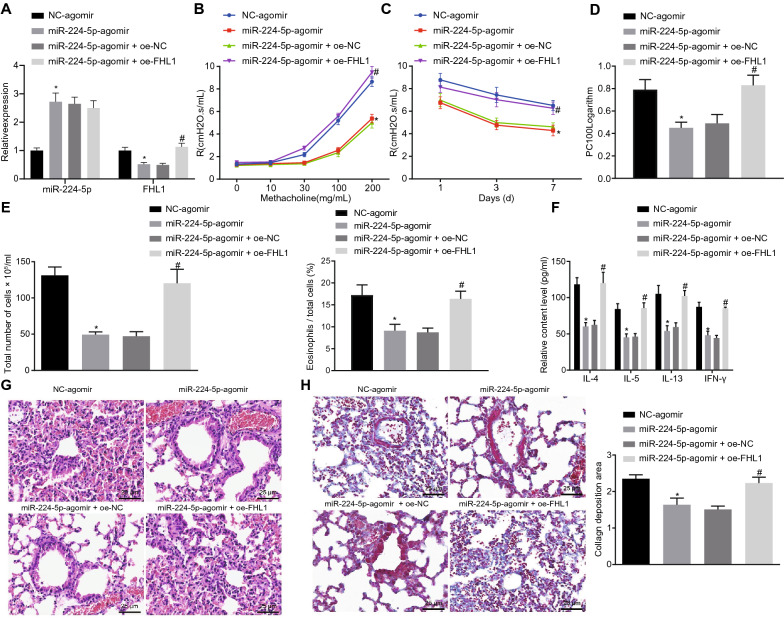
MiR-224-5p reduces airway inflammation in asthmatic mice through FHL1 suppression. Asthmatic mice were treated with lentivirus harboring miR-224-5p-agomir/NC-agomir alone or in combination with lentivirus harboring oe-FHL1/oe-NC. **A** Expression of miR-224-5p and FHL1 in lung tissues of asthmatic mice determined by RT-qPCR. **B** The airway resistance in asthmatic mice on the 1st day. **C** The airway resistance in asthmatic mice at various time points when the dosage of Mch is 200 mg/mL. **D**, The airway responsiveness in asthmatic mice. **E** The number of total cells (the left) and percentage of eosinophils in BALF of asthmatic mice. **F** Levels of IL-4, IL-5, IL-13 and IFN-γ in BALF supernatant of asthmatic mice measured by ELISA. **G** HE staining images of lung tissues from asthmatic mice. **H** Masson’s trichrome staining images of lung tissues and collagen deposition area from asthmatic mice. * *p* < 0.05 compared with asthmatic mice treated with lentivirus harboring NC-agomir. # *p* < 0.05 compared with asthmatic mice treated with lentiviruses expressing miR-224-5p-agomir and oe-NC. n = 10 mice for each treatment

miR-224-5p was overexpressed in asthmatic mice to characterize whether the function of miR-224-5p in asthma was achieved by targeting FHL1. We observed a reduction in FHL1 expression and an increase in miR-224-5p expression in lung tissues of asthmatic mice treated with miR-224-5p-agomir, while oe-FHL1 treatment restored FHL1 expression (Fig. 5A).

In response to Mch at a dosage over 30 mg/mL or a dosage of 200 mg/mL (Fig. 5B–D), ectopic expression of miR-224-5p weakened airway resistance and airway responsiveness, while restoration of FHL1 reversed this effect.

The percentage of eosinophils was reduced when miR-224-5p was overexpressed, which was reversed following further upregulation of FHL1 (Fig. 5E). Moreover, miR-224-5p overexpression reduced the number of total cells and the percentage of eosinophils in the BALF and the secretion of inflammatory factors (IL-4, IL-5, IL-13, and IFN-γ) in BALF supernatant of asthmatic mice, which were negated by the further FHL1 overexpression (Fig. 5E–F).

According to the results of HE staining and Masson’s trichrome staining (Fig. 5G–H), asthmatic mice treated with NC-agomir or the combination of miR-224-5p-agomir and oe-FHL1 displayed a large amount of inflammatory cell infiltration (mainly eosinophils and lymphocytes) around the airway mucosa and blood vessel wall. Meanwhile, we also observed thickened airway wall and smooth muscle, increased folds of the mucosal epithelium, narrowed lumen, some lumen blocked by mucus plugs, and increased staining area around bronchus and content of lung collagen. Besides, the asthmatic mice treated with miR-224-5p-agomir exhibited orderly arranged epithelium of airway mucosa, slight damage of lung tissues, and reduced thickness of bronchial wall and content of collagen.

Altogether, our data indicated that overexpression of miR-224-5p ameliorated airway inflammation and lung tissue damage in the asthmatic mice by inhibiting FHL1.

### Silencing of FHL1 represses the MAPK pathway in vivo

**Fig. 6:**
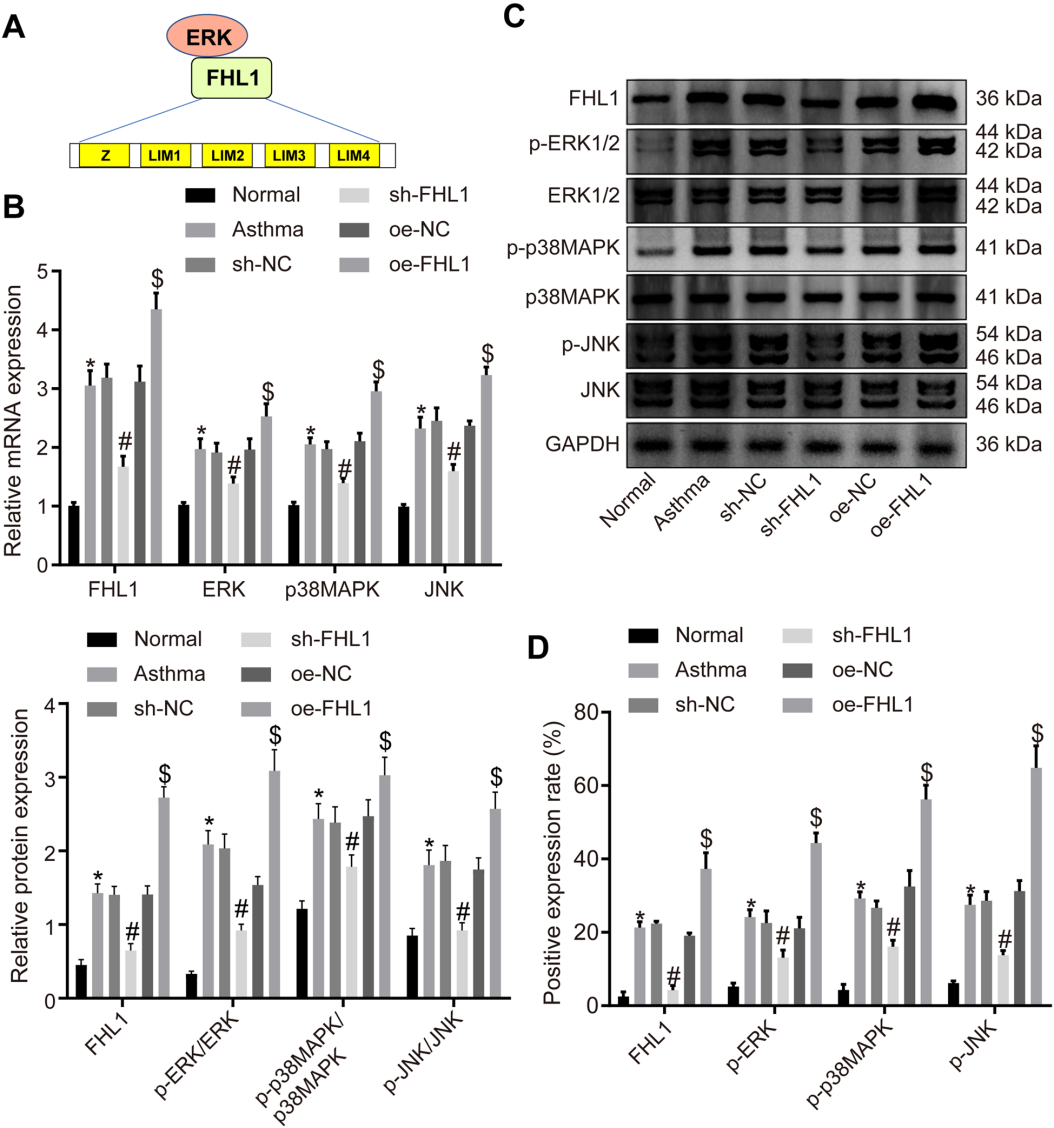
Silencing of FHL1 blocks the MAPK pathway in vivo. Normal mice served as the control and asthmatic mice were treated with lentivirus harboring oe-FHL1/oe-NC or sh-FHL1/sh-NC. **A** Schematic diagram of the binding of MAPK pathway protein ERK to the LIM domain of FHL1, which is composed of four LIM domains (lim1-4) and an additional N-terminal half LIM domain (z). **B** mRNA levels of FHL1, ERK, p38MAPK and JNK in lung tissues of asthmatic mice determined by RT-qPCR. **C** Protein levels of FHL1, ratio of p-ERK/ERK, ratio of p-p38MAPK/p38MAPK, and ratio of p-JNK/JNK in lung tissues of asthmatic mice measured by Western blot analysis. **D** Immunohistochemical analysis of protein expression of p-ERK, p-p38MAPK, and p-JNK in lung tissues of asthmatic mice. n = 10 mice for each treatment. * *p* < 0.05 compared with normal mice. # *p* < 0.05 compared with asthmatic mice treated with lentivirus harboring sh-NC. $ *p* < 0.05 compared with asthmatic mice treated with lentivirus harboring oe-NC

Evidence exists reporting that activation of the MAPK pathway is associated with asthma [29, 30] and FHL1 can regulate the MAPK pathway [18]. The schematic diagram of the binding of MAPK pathway protein ERK to the LIM domain of FHL1 is shown in Fig. 6A. Therefore, we speculated that aberrant expression of FHL1 may mediate the MAPK pathway in asthma.

We confirmed that the mRNA levels of FHL1, ERK, p38MAPK and JNK as well as the protein levels of FHL1, ratio of p-ERK/ERK, ratio of p-p38MAPK/p38MAPK, and ratio of p-JNK/JNK were increased in lung tissues of asthmatic mice relative to normal mice. overexpression of FHL1 further enhanced the increases yet FHL1 knockdown reversed these increases (Fig. 6B–D, Additional file 3:Fig. S3A).

Hence, downregulation of FHL1 could block the activation of the MAPK pathway in asthma.

### MiR-224-5p impairs ASMC proliferation and induces their apoptosis by suppressing FHL1

**Fig. 7:**
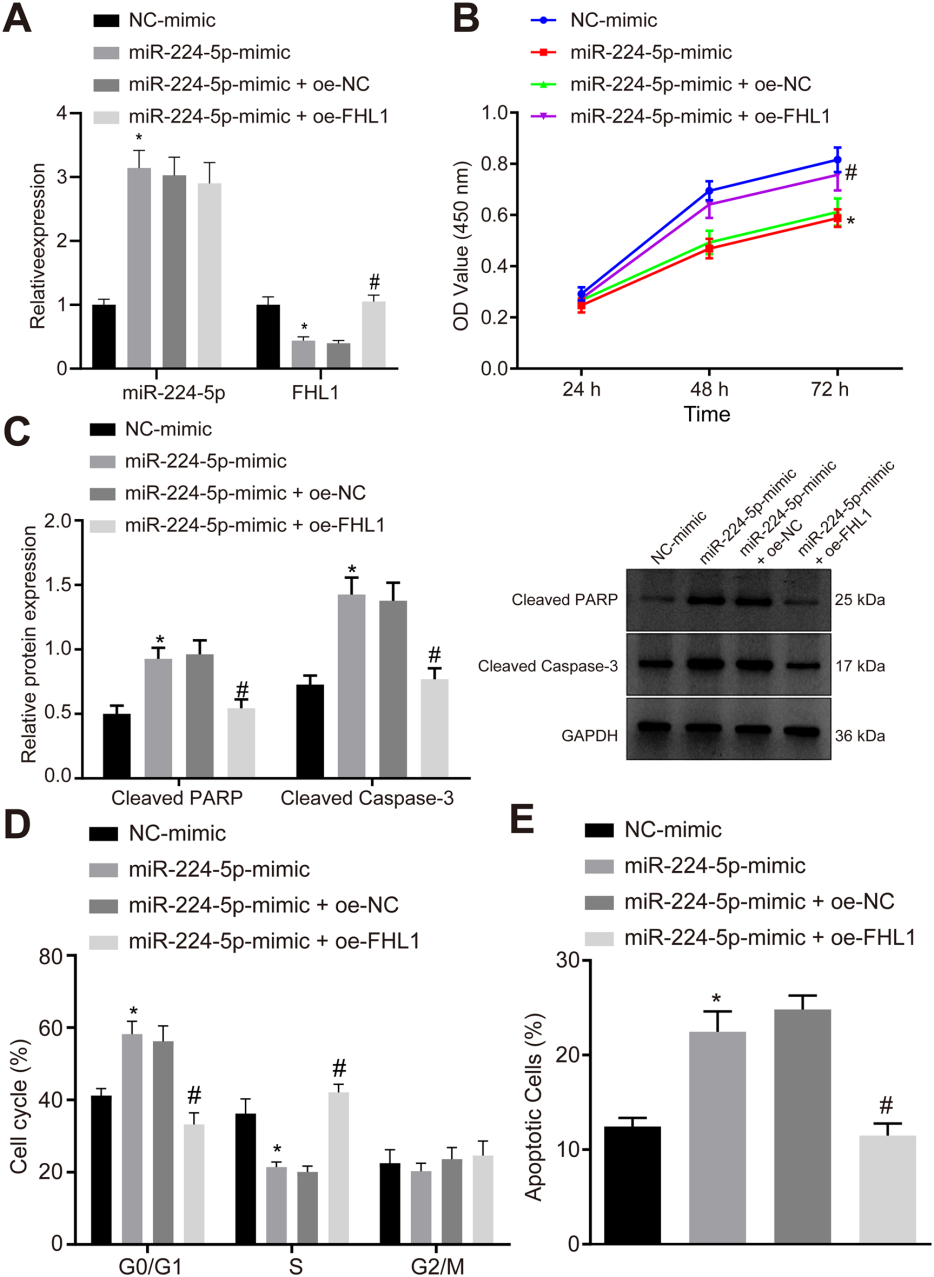
MiR-224-5p inhibits proliferation and enhances apoptosis of ASMCs by repressing FHL1. ASMCs were transduced with miR-224-5p mimic/NC-mimic or in combination with oe-FHL1/oe-NC. **A** Expression of miR-224-5p and FHL1 in ASMCs determined by RT-qPCR. **B** ASMC proliferation examined by MTT assay. **C** Protein levels of cleaved PARP and cleaved caspase-3 in ASMCs measured by Western blot analysis. **D** Flow cytometric data showing cell cycle distribution. **E** Flow cytometric data showing cell apoptosis. * *p* < 0.05 compared with ASMCs transduced with NC-mimic. # *p* < 0.05 compared with ASMCs transduced with miR-224-5p-mimic + oe-NC. Cell experiments were performed in triplicate

The expression of miR-224-5p and FHL1 was manipulated in ASMCs to explore their impacts on the biological functions of ASMCs. RT-qPCR suggested that miR-224-5p expression was upregulated while FHL1 expression was downregulated in miR-224-5p mimic-transfected ASMCs. By contrast, co-overexpression of miR-224-5p and FHL1 resulted in enhanced FHL1 expression relative to miR-224-5p upregulation alone (Fig. 7A).

MTT assay, flow cytometry, and Western blot analysis subsequently exhibited inhibited viability of ASMCs, more cells arrested at the G1/G0 phase, fewer cells arrested at S phase, promoted cell apoptosis, and elevated proteins levels of cleaved PARP and cleaved caspase-3 in the ASMCs from asthmatic mice in response to restored miR-224-5p, which were abolished after simultaneous overexpression of FHL1 (Fig. 7B–E).

Altogether, miR-224-5p could inhibit ASMC proliferation and facilitate their apoptosis by repressing FHL1 expression in vitro.

### MiR-224-5p disrupts the activation of MAPK pathway by downregulating FHL1 in vivo

**Fig. 8:**
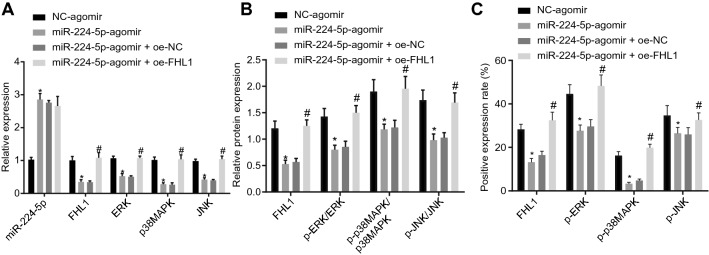
MiR-224-5p blocks the MAPK pathway activation by inhibiting FHL1 in vivo. Asthmatic mice were treated with lentivirus harboring miR-224-5p-agomir/NC-agomir or in combination with lentivirus harboring oe-FHL1/oe-NC. **A** Expression of miR-224-5p, FHL1, ERK, p38MAPK, and JNK in lung tissues of asthmatic mice determined by RT-qPCR. **B** Protein levels of FHL1, ratio of p-ERK/ERK, ratio of p-p38MAPK/p38MAPK, and ratio of p-JNK/JNK in lung tissues of asthmatic mice determined by Western blot analysis. **C** Protein expression of p-ERK, p38MAPK, and p-JNK in lung tissues of asthmatic mice detected by immunohistochemical analysis. n = 10 mice for each treatment. * *p* < 0.05 compared with asthmatic mice treated with lentivirus harboring NC-agomir. # *p* < 0.05 compared with asthmatic mice treated with lentivirus harboring miR-224-5p-agomir + oe-NC

Finally, we determined the expression of the MAPK pathway-related molecules in the lung tissues of asthmatic mice. It was confirmed that mRNA levels of FHL1, ERK, p38MAPK, and JNK as well as the protein levels of FHL1, ratio of p-ERK/ERK, ratio of p-p38MAPK/p38MAPK, and ratio of p-JNK/JNK were lowered following miR-224-5p upregulation, all of which were reversed when FHL1 was simultaneously restored (Fig. 8A,B–C, Additional file 3: Fig. S3B). Hence, miR-224-5p could block the activation of the MAPK pathway through suppression of FHL1 in vivo*.*

## Discussion

miRNAs have been identified as promising biomarkers in airway inflammation and the function of ASMCs in asthma [31, 32]. Herein, our study is conducted to explore the effects of miR-224-5p on airway inflammation in asthmatic mice and proliferation of ASMCs with the involvement of the FHL1-mediated MAPK pathway. Collectively, the results demonstrated an inhibitory property of miR-224-5p on airway inflammation and ASMC proliferation via disruption of the FHL1-mediated MAPK pathway.

Our study found that FHL1 was highly expressed in the lung tissues of asthmatic mic, and downregulation of FHL1 alleviated airway inflammation, inhibited ASMC proliferation, and promoted cell apoptosis in asthmatic mice. This finding corroborates that of a previous study, which has highlighted that FHL1 restricts the host adaptive immune response, and then triggers influenza A virus-induced lung inflammation [33]. FHL2, another FHL protein family member, serves as a new target for treating asthma, and silencing of FHL2 suppresses airway inflammation in OVA-induced asthmatic mice [34]. Another study has also demonstrated that FHL1 is a prominently upregulated protein in lung tissues from patients with idiopathic pulmonary arterial hypertension and its overexpression enhances migration and proliferation of pulmonary artery SMCs [35]. FHL1 knockdown arrested pulmonary artery SMCs at the G1 phase and dampened cell proliferation in hypoxia-induced pulmonary hypertension [12]. These evidences support the roles of FHL1 in the inhibition of airway inflammation and ASMC proliferation in asthmatic mice.

Of note, the mechanistic data revealed that FHL1 was targeted by miR-224-5p in asthma, and miR-224-5p was downregulated in lung tissues of asthmatic mouse. The functional experiments of our work highlighted that miR-224-5p alleviated airway inflammation, repressed proliferation and stimulated apoptosis of ASMCs in asthmatic mice. In line with our results, downregulation of miR-224 has also been previously observed in asthmatic patients [23]. The anti-asthmatic effect of miR-224-5p in OVA-induced experimental asthma has been documented to be achieved by restricting pro-inflammatory TLR2 expression [24]. The infiltration of inflammatory cells in OVA-induced experimental allergic rhinitis was appreciably diminished by miR-224 agomir, and this effect was associated with targeted inhibition of CDK9 [36]. The forced expression of miR-224-5p exerted protective effect against airway inflammation in asthmatic mice by targeting FHL1, and overexpressed FHL1 could offset its effect.

Furthermore, miR-224-5p has been documented to inactivate the MAPK/ERK pathway in rats with hypothyroidism [37]. MAPK pathway is correlated with some inflammatory responses of patients with asthma, such as the release of inflammatory proteins TGF-β1 and IL-13 [38]. Suppression of the MAPK pathway activation could curb ASMC proliferation, migration and subsequent airway remodeling in asthma [39]. Consistently, our data validated the suppression of miR-224-5p on the MAPK pathway activation, and further offered evidence reporting the implication of FHL1. Restored miR-224-5p blocked the MAPK pathway to ameliorate airway inflammation in asthmatic mice, while ectopic expressed FHL1 negated its effect. FHL1 is a biomechanical stress sensor that mediates the MAPK-activated pathway [18, 40], thus the miR-224-5p-impaired FHL1 expression could restrict the MAPK signaling. Therefore, we reasoned that miR-224-5p could ameliorate airway inflammation, repress proliferation of ASMCs by blocking activation of FHL1-dependent MAPK pathway.

## Conclusions

Overall, the present study demonstrated that miR-224-5p may act as an anti-asthma miRNA by suppressing airway inflammation in the OVA-induced asthmatic mice. Our study revealed that miR-224-5p could suppress ASMC-secreted inflammatory factors, including IL-4, IL-5, IL-13 and IFN-γ. Additionally, miR-224-5p repressed proliferation and promoted apoptosis of ASMCs by inhibiting FHL1-mediated MAPK pathway activation. Therefore, it is promising to develop therapeutic strategies targeting miR-224-5p for clinical use in the treatment of asthma. Moreover, the inner mechanisms of miR-224-5p and FHL1 in clinical tissue samples of asthma and the effect of miR-224-5p on mild or chronic asthma mouse models should be further explored and confirmed in the future.

## Supplementary Information

**Additional file 1: Figure S1.** Schematic map of the function miR-224-5p in asthma. miR-224-5p inhibits FHL1 expression and then blocks the MAPK pathway activation, thus suppressing airway inflammation in asthmatic mice and ASMC proliferation while promoting ASMC apoptosis.

**Additional file 2: Figure S2. In vivo **experiment flow chart.

**Additional file 3: Figure S3.** Representative experimental images of immunohistochemical analysis, corresponding to the quantitative data in Figure 6C (A) and 8C (B).

**Additional file 4: Table S1.** Primer sequences for RT-qPCR

## Data Availability

The datasets generated and/or analysed during the current study are available from the corresponding author on reasonable request.

## Abbreviations

miRNAs: MicroRNAs
TLR2: Toll-like receptor 2
FC: Fold change
sh: Short hairpin RNA
miR-224-5p: MicroRNA-224-5p
FHL1: Four-and-a-half LIM protein 1
GEO: Gene expression omnibus
NCBI: National center for biotechnology information
JNK: C-Jun N-terminal kinase
MAPK: Mitogen-activated protein kinase
ASMCs: Airway smooth muscle cells
VEGF: Vascular endothelial growth factor
OVA: Ovalbumin; PBS: phosphate-buffered saline
BALF: Bronchoalveolar lavage fluid
HE: Hematoxylin–eosin
oe: Overexpression
NC: Negative control
Mch: Methacholine0
RI: Inspiratory resistance
H_2_O_2_: Hydrogen peroxide
p: Phosphorylated
IgG: Immunoglobulin G
IFN-γ: Interferon-γ
IL: Interleukin
DAB, 3: 3'-Diaminobenzidine
ELISA: Enzyme linked immunosorbent assay
NC: Negative control
OD: Optical density
DMEM: Dulbecco's modified Eagle's medium
FBS: Fetal bovine serum
EDTA: Ethylene diamine tetraacetic acid
SP: Streptavidin–biotin-peroxidase
EDTA: Ethylenediamine tetraacetic acid
mut: Mutant
3’-UTR: 3’-Untranslated region
RT-qPCR: Reverse transcription-quantitative polymerase chain reaction
cDNA: Complementary DNA
GAPDH: Glyceraldehyde-3-phosphate dehydrogenase
ERK: Extracellular signal-regulated kinase
BCA: Bicinchoninic acid
RIPA: Radioimmunoprecipitation assay
HRP: Horseradish peroxidase
ECL: Enhanced chemiluminescent
PVDF: Polyvinylidene fluoride
BSA: Bovine serum albumin
MTT: 3-(4,5-Dimethylthiazol-2-yl)-2, 5-diphenyltetrazolium bromide
FITC: Fluorescein isothiocyanate
PI: Propidium iodide
HEPES: 4-(2-Hydroxyethyl)-1-piperazineëthanesulfonic acid
DMSO: Dimethyl sulfoxide
ANOVA: Analysis of variance
WT: Wild type
