# Supplementary figures and images for: Anti-asthmatic miR-224-5p inhibits the FHL1/MAPK pathway to repress airway smooth muscle cell proliferation in a murine model of asthma-like airway inflammation
Source: Allergy Asthma Clin Immunol. 2022 Oct 2;18:88. doi: 10.1186/s13223-022-00724-9 (PMC9526920; doi:10.1186/s13223-022-00724-9)

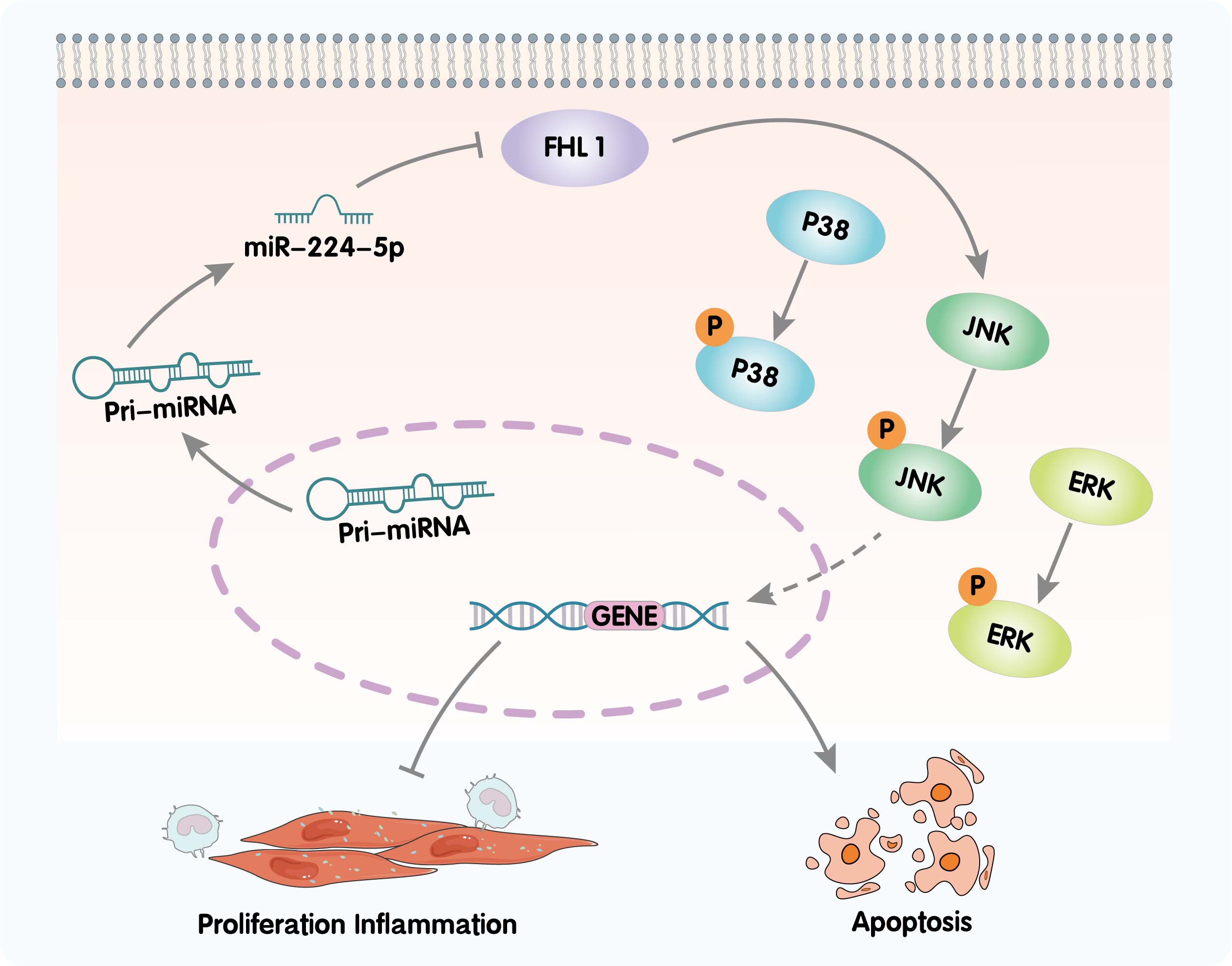

Supplement: Supplementary file 1 — Additional file 1: Figure S1. Schematic map of the function miR-224-5p in asthma. miR-224-5p inhibits FHL1 expression and then blocks the MAPK pathway activation, thus suppressing airway inflammation in asthmatic mice and ASMC proliferation while promoting ASMC apoptosis. [file 13223_2022_724_MOESM1_ESM.jpg]

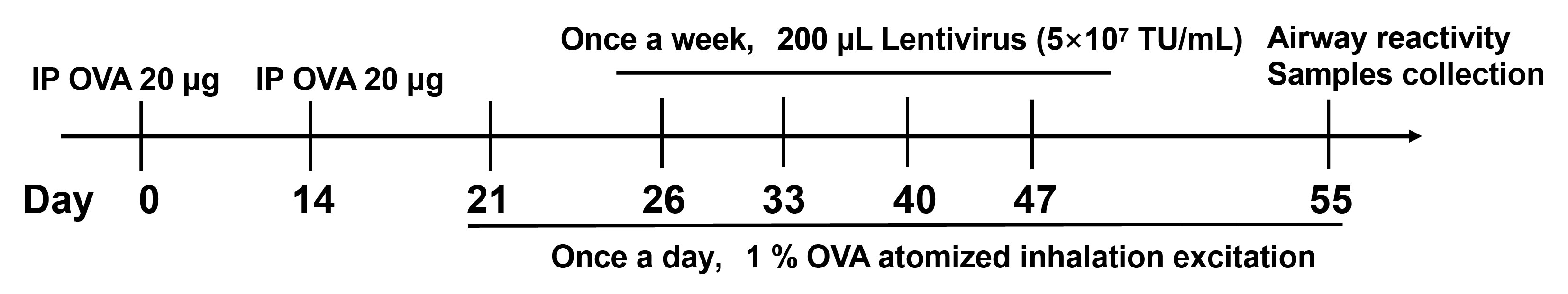

Supplement: Supplementary file 2 — Additional file 2: Figure S2. In vivo experiment flow chart. [file 13223_2022_724_MOESM2_ESM.jpg]

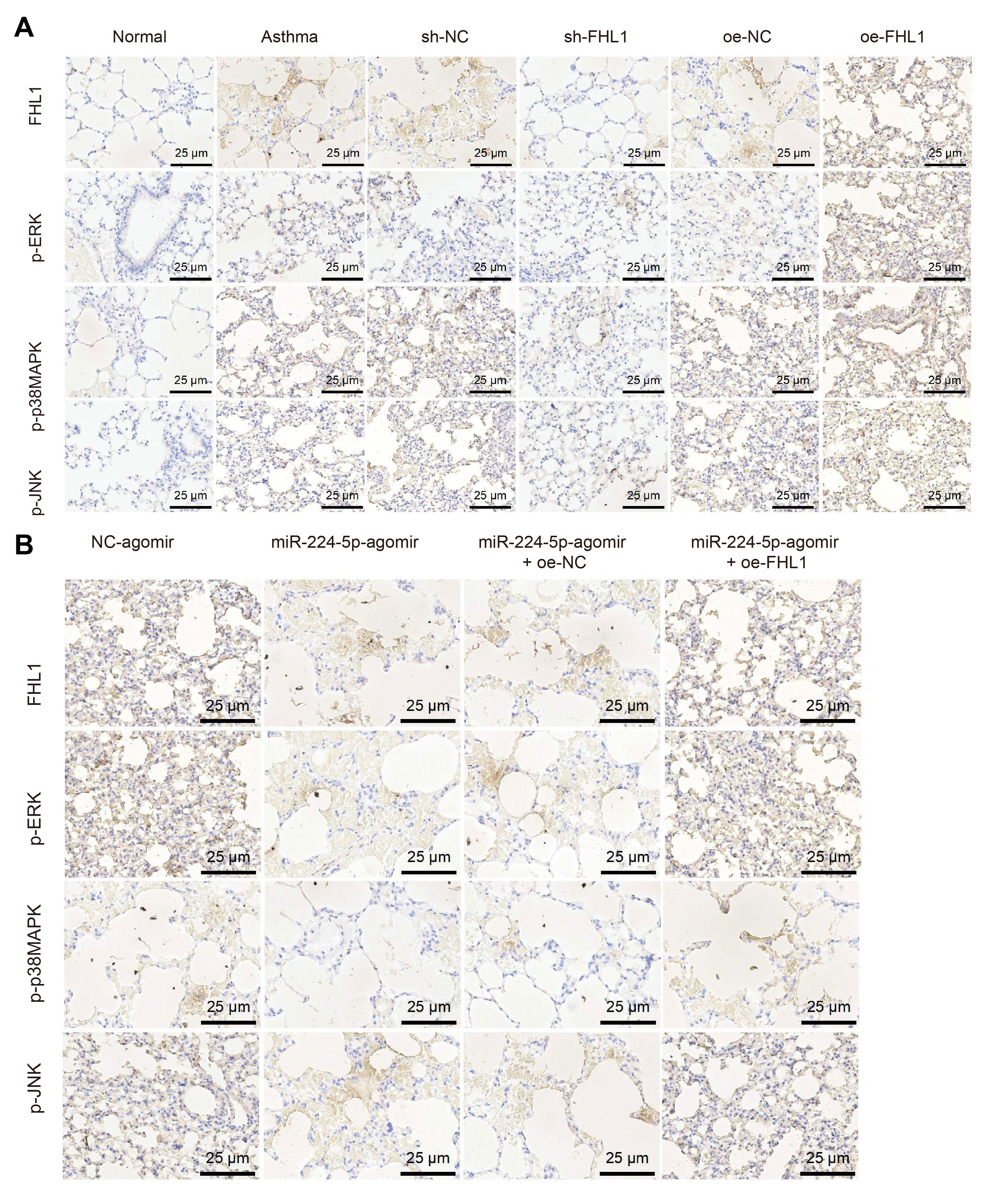

Supplement: Supplementary file 3 — Additional file 3: Figure S3. Representative experimental images of immunohistochemical analysis, corresponding to the quantitative data in Figure 6C (A) and 8C (B). [file 13223_2022_724_MOESM3_ESM.jpg]
