# Supplementary material for: Anti-asthmatic miR-224-5p inhibits the FHL1/MAPK pathway to repress airway smooth muscle cell proliferation in a murine model of asthma-like airway inflammation
Source: Allergy Asthma Clin Immunol. 2022 Oct 2;18:88. doi: 10.1186/s13223-022-00724-9 (PMC9526920; doi:10.1186/s13223-022-00724-9)
Supplement: Supplementary file 4 — Additional file 4: Table S1. Primer sequences for RT-qPCR [file 13223_2022_724_MOESM4_ESM.docx]

**Table S1** Primer sequences for RT-qPCR

| Gene | Sequence（5'-3'） |
| --- | --- |
| miR-224-5p | Forward: TAAGTCACTAGTGGTTCCGTT |
|  | Reverse: Universal reverse primer |
| FHL1 | Forward: TAAGAATCGCTACTGGCACGA |
|  | Reverse: AATGGCCTTGAAGCACCCTTT |
| ERK | Forward: CTCTCATTTGAGGACAGGCA |
|  | Reverse: ATGTGGCATGCAGTGTAGGT |
| p38MAPK | Forward: CGCTGCTGCCGCTGGAAGAT |
|  | Reverse: TTTGGCGTGAATGATGGACT |
| JNK | Forward: GTGGGGTATGCCCAAGAGG |
|  | Reverse: GCCATAAAGCCCAGATAGAGC |
| GAPDH | Forward: CCCTTAAGAGGGATGCTGCC |
|  | Reverse: ACTGTGCCGTTGAATTTGCC |
| U6 | Forward: CTCGCTTCGGCAGCACA |
|  | Reverse: Universal reverse primer |

Note: miR-224-5p, microRNA-224-5p; FHL1, four-and-a-half LIM domain protein 1; ERK, extracellular signal-regulated kinase; p38MAPK, p38 mitogen-activated protein kinase; JNK, c-Jun N-terminal kinase; GAPDH, glyceraldehyde-3-phosphate dehydrogenase; RT-qPCR, reverse transcription-quantitative polymerase chain reaction.
